# Early Adoption of Longitudinal Surveillance for SARS-CoV-2 among Staff in Long-Term Care Facilities: Prevalence, Virologic and Sequence Analysis

**DOI:** 10.1128/Spectrum.01003-21

**Published:** 2021-11-10

**Authors:** Emily N. Gallichotte, Kendra M. Quicke, Nicole R. Sexton, Emily Fitzmeyer, Michael C. Young, Ashley J. Janich, Karen Dobos, Kristy L. Pabilonia, Gregory Gahm, Elizabeth J. Carlton, Gregory D. Ebel, Nicole Ehrhart

**Affiliations:** a Colorado State University, Ft. Collins, Colorado, USA; b University of Colorado Medical Center, Aurora, Colorado, USA; c Vivage Senior Living, Denver, Colorado, USA; d University of Colorado Anschutz, Aurora, Colorado, USA; Houston Methodist Hospital

**Keywords:** COVID-19, long-term care, SARS-CoV-2, coronavirus, epidemiology, infectious disease

## Abstract

Severe acute respiratory syndrome coronavirus 2 (SARS-CoV-2) emerged in 2019 and has become a major global pathogen in an astonishingly short period of time. The emergence of SARS-CoV-2 has been notable due to its impacts on residents in long-term care facilities (LTCFs). LTCF residents tend to possess several risk factors for severe outcomes of SARS-CoV-2 infection, including advanced age and the presence of comorbidities. Indeed, residents of LTCFs represent approximately 40% of SARS-CoV-2 deaths in the United States. Few studies have focused on the prevalence and transmission dynamics of SARS-CoV-2 among LTCF staff during the early months of the pandemic, prior to mandated surveillance testing. To assess the prevalence and incidence of SARS-CoV-2 among LTCF staff, characterize the extent of asymptomatic infections, and investigate the genomic epidemiology of the virus within these settings, we sampled staff for 8 to 11 weeks at six LTCFs with nasopharyngeal swabs from March through June of 2020. We determined the presence and levels of viral RNA and infectious virus and sequenced 54 nearly complete genomes. Our data revealed that over 50% of infections were asymptomatic/mildly symptomatic and that there was a strongly significant relationship between viral RNA (vRNA) and infectious virus, prolonged infections, and persistent vRNA (4+ weeks) in a subset of individuals, and declining incidence over time. Our data suggest that asymptomatic SARS-CoV-2-infected LTCF staff contributed to virus persistence and transmission within the workplace during the early pandemic period. Genetic epidemiology data generated from samples collected during this period support that SARS-CoV-2 was commonly spread between staff within an LTCF and that multiple-introduction events were less common.

**IMPORTANCE** Our work comprises unique data on the characteristics of SARS-CoV-2 dynamics among staff working at LTCFs in the early months of the SARS-CoV-2 pandemic prior to mandated staff surveillance testing. During this time period, LTCF residents were largely sheltering-in-place. Given that staff were able to leave and return daily and could therefore be a continued source of imported or exported infection, we performed weekly SARS-CoV-2 PCR on nasal swab samples collected from this population. There are limited data from the early months of the pandemic comprising longitudinal surveillance of staff at LTCFs. Our data reveal the surprisingly high level of asymptomatic/presymptomatic infections within this cohort during the early months of the pandemic and show genetic epidemiological analyses that add novel insights into both the origin and transmission of SARS-CoV-2 within LTCFs.

## INTRODUCTION

The highly infectious severe acute respiratory syndrome coronavirus 2 (SARS-CoV-2) threatened the stability of health care systems around the world. Long-term care facilities (LTCFs), due to their communal nature, the limited mobility of their inhabitants, and the propensity of residents to have underlying health conditions, were severely affected across the United States (1), resulting in disproportionally high morbidity and mortality among residents in LTCFs. As of 30 June 2021, the Centers for Medicare and Medicaid Services reported over 184,000 deaths due to coronavirus disease 2019 (COVID-19) in U.S. LTCFs, representing over 31% of COVID-19-related deaths (2, 3). In the United States, the first recorded SARS-CoV-2 outbreak occurred in an LTCF in Washington as early as February 2020 (4). Since then, every state has recorded outbreaks in LTCFs, and in 14 states, LTCF deaths account for over 50% of all COVID-19 deaths (3). The high mortality associated with SARS-CoV-2 infection within LTCFs is principally due to the risk profiles of residents residing in communal care settings, including advanced age and preexisting comorbidities like heart disease and diabetes mellitus (5–7).

Accordingly, strategies to mitigate SARS-CoV-2 transmission to LTCF residents have included restricting visitation, cessation of group activities and dining, and confinement to individual living quarters (8, 9). While LTCF residents have been largely isolated from external visitation, staff were permitted contact provided they had passed a daily screening process to assess for fever, COVID-19 respiratory symptoms, or known exposure (10). These staff have the potential to import the virus into facilities, resulting in spread to residents, other workers, and back to the outside community (1). While symptom screening can reduce virus spread, a significant fraction of individuals infected with SARS-CoV-2 have a lengthy latency period prior to exhibiting COVID-19 symptoms, and many remain asymptomatic throughout the course of infection (11–16). Therefore, we theorized that presymptomatic, asymptomatic, and mildly symptomatic LTCF staff were a potential source of transmission within LTCFs and were a critical population to study to better understand mitigation strategies for optimizing infection prevention strategies (13, 14, 17–21).

While there are several studies characterizing SARS-CoV-2 infection within LTCF residents, there have been limited studies focused on the longitudinal surveillance of LTCF staff (22). To evaluate the impact of staff on virus introduction into LTCFs, we tested staff at six Colorado LTCFs for SARS-CoV-2 from March to June of 2020. Staff were enrolled and sampled by nasopharyngeal (NP) swab weekly for 8 to 11 consecutive weeks. Samples were assayed for virus by reverse transcription-quantitative PCR (qRT-PCR) and plaque assay, and individuals with evidence of infection were instructed to self-quarantine for 10 days. Using data on staff infection, the site-specific prevalences at study onset and incidence rates over time were calculated. Viral genomes were sequenced to assess viral genetic diversity within and between LTCFs.

Our results document a surprising degree of asymptomatic/mildly symptomatic infection among apparently healthy staff and extreme variation in SARS-CoV-2 prevalence and incidence between different facilities, similar to what has been observed at other LTCFs (13, 14, 17, 20). We documented a range of infection courses, including acute (1 week), prolonged (4+ weeks), and recrudescent infections. Sequencing studies lend support to the observation that transmission may occur within LTCFs and, combined with the epidemiologic and other data provided here, highlight the importance of testing and removing virus-positive workers in order to protect vulnerable LTCF residents. Data obtained from longitudinal surveillance studies provide crucial information about infectious disease transmission dynamics within complex workforces and inform best practices for preventing or mitigating COVID-19 outbreaks within LTCFs.

## RESULTS

### Cohort characteristics.

From 26 March to 23 June 2020, we tested 544 staff from six LTCFs (Table 1). Participation was voluntary, and between 50 and 80% of eligible staff enrolled in our study. Weekly participation of enrolled staff varied across facilities; however, over 50% tested for at least 7 weeks at each site (Fig. S1 in the supplemental material). Of these participants, 91 (16.7%) apparently healthy staff tested positive for SARS-CoV-2 viral RNA (vRNA) at least once during the study. We tested 3,754 samples in total, of which 179 were positive for vRNA (4.77% of the total samples).

**TABLE 1:** Positive test results for Colorado LTCF cohort by site

Site	No. (%) of individuals in group^*a*^	
	All participants (*n* = 544)	vRNA^+^ participants (*n* = 91)
A	100 (18)	0 (0)
B	108 (20)	8 (9)
C	51 (9)	10 (11)
D	128 (24)	54 (59)
E	76 (14)	14 (15)
F	81 (15)	5 (5)

a The total numbers of samples tested included 3,591 NP swab samples for all participants, 179 NP swab samples for vRNA^+^ participants, 163 saliva samples all participants, and 0 saliva samples for vRNA^+^ participants.

### Viral loads, prevalences, and incidence rates varied across LTCFs.

**FIG 1:**
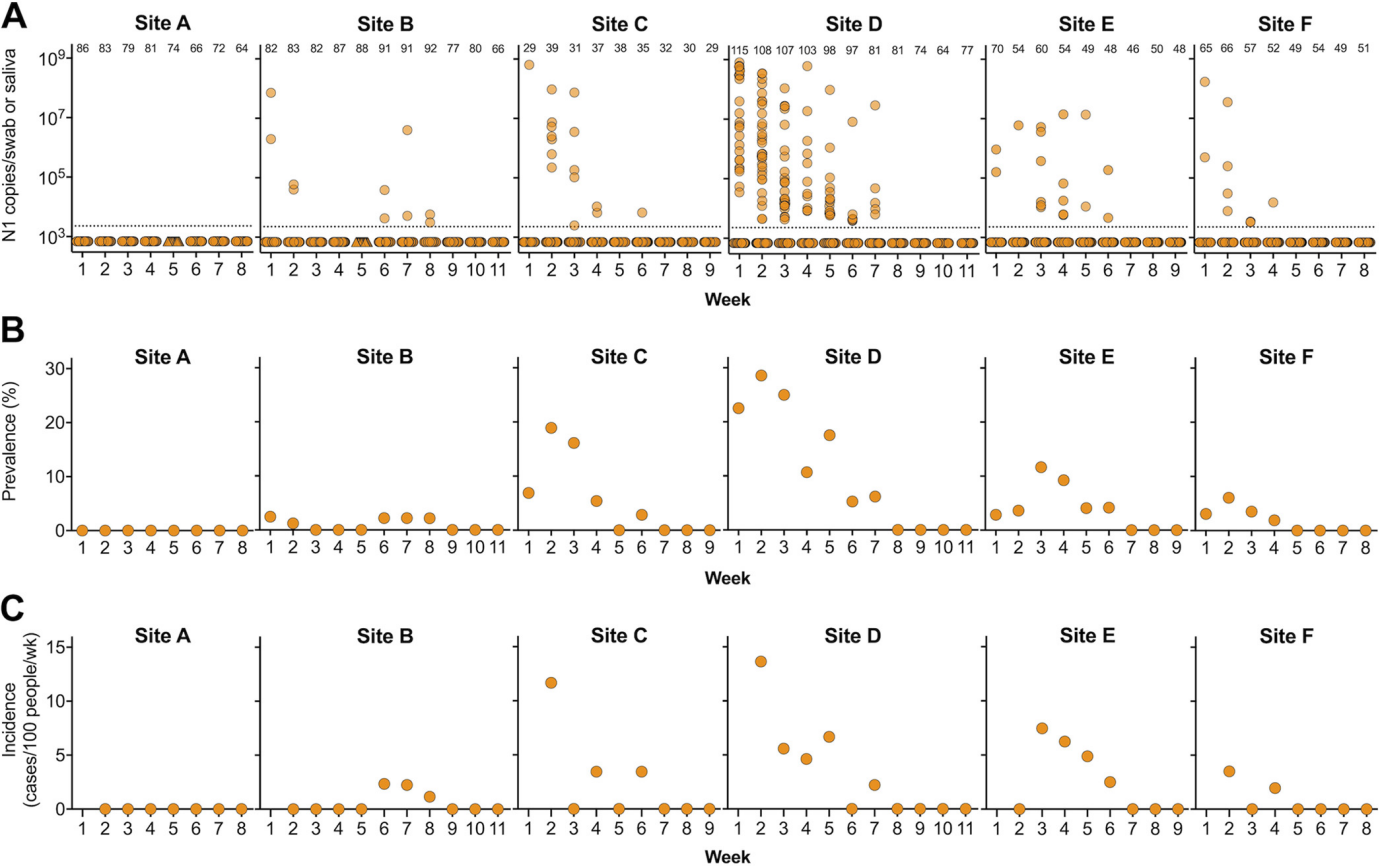
SARS-CoV-2 infections in six Colorado LTCFs. (A) SARS-CoV-2 N1 vRNA levels in nasopharyngeal swab (circles) or saliva (triangles) samples. Saliva was only sampled a single time (week 5) at two sites (A and B) due to nasopharyngeal swab shortages. *y* axis represents N1 copy number per swab or saliva sample. Dotted line indicates limit of detection. Numbers across the top indicate number of samples tested each week. (B) Prevalence of SARS-CoV-2 each week at each site (percentage of samples with detectable N1 vRNA out of total number tested). (C) Incident cases were defined as individuals who tested positive for N1 vRNA for the first time and had tested negative for infection 1 or 2 weeks prior. Not shown are prevalent infections among workers tested for the first time in week 2.

The viral RNA levels and the prevalence of vRNA-positive (vRNA^+^) swabs varied each week by site (Fig. 1A and B). None of the saliva samples (Fig. 1A, triangles) tested positive; however, this was likely due to low infection prevalence at the facilities the week saliva testing was performed. Staff at site A remained uninfected throughout the 8-week study period, whereas 31% of individuals at site D were infected in week two. All sites showed a decline in SARS-CoV-2 prevalence over the course of the study (Fig. 1B). SARS-CoV-2 incidence also varied across sites (Fig. 1C). At site D, which had the highest SARS-CoV-2 prevalence, the initial incidence was also high (13.6 cases per 100 person-weeks) but declined over time. At sites C and F, the incidence reached zero by week 3. In the following week, however, both sites C and F experienced a small number of incident cases, suggesting a new reintroduction of the virus. Sites B and E, which had low prevalences in week 1, saw an increase in cases. At site B, incident infections were detected after 3 weeks.

Importantly, sites D, E, and F all reported SARS-CoV-2 outbreaks to the Colorado public health department, coinciding with our study period. During our surveillance testing of staff, these sites reported 29, 3, and 4 resident infections at sites D, E, and F, respectively. The high resident infection rate at site D matched the high SARS-CoV-2 prevalence of staff (Fig. 1D). All facilities were under public health orders to be masked, including staff that did not have direct patient contact. Actual compliance with mask and other personal protective equipment (PPE) orders might have varied across facilities, leading to higher infection rates in staff and residents.

Infections were observed in all job classes, including those with typically high patient contact (e.g., nursing) and low patient contact (e.g., maintenance) (Table 2). The highest odds ratios for infection occurred in housekeeping, nursing, and staff in jobs classified as “other,” while the lowest were in administration, therapy, and dietary staff (Table 2).

**TABLE 2:** Analysis of infections in LTCF staff by job code

Job code	No. tested	% positive	OR (95% CI)^*a*^	
			Unadjusted	Adjusted
Administration	53	11.3	1.00 (Ref)	1.00 (Ref)
Nursing	180	24.4	2.53 (1.01, 6.33)	2.79 (1.07, 7.32)
Housekeeping	96	14.6	1.34 (0.48, 3.71)	4.69 (1.39, 15.84)
Dietary	36	19.4	1.89 (0.58, 6.18)	1.55 (0.45, 5.34)
Therapy	24	4.2	0.34 (0.04, 3.00)	0.47 (0.05, 4.45)
Other^*b*^	46	34.8	4.18 (1.47, 11.87)	4.91 (1.61, 14.97)

a The analysis looks at the percentage of workers that tested positive at least once during the study period. Analysis is limited to the five sites where SARS-CoV-2 was detected (B, C, D, E, and F). Unadjusted odds ratios were estimated using logistic regression, and adjusted analyses included a dummy variable for site. OR, odds ratio; Ref, reference job code used for odds-ratio analyses.

b Other jobs include physician/provider, maintenance, social services, transport, and activities.

### Relationship between viral RNAs and infectious virus in nasopharyngeal swabs.

**FIG 2:**
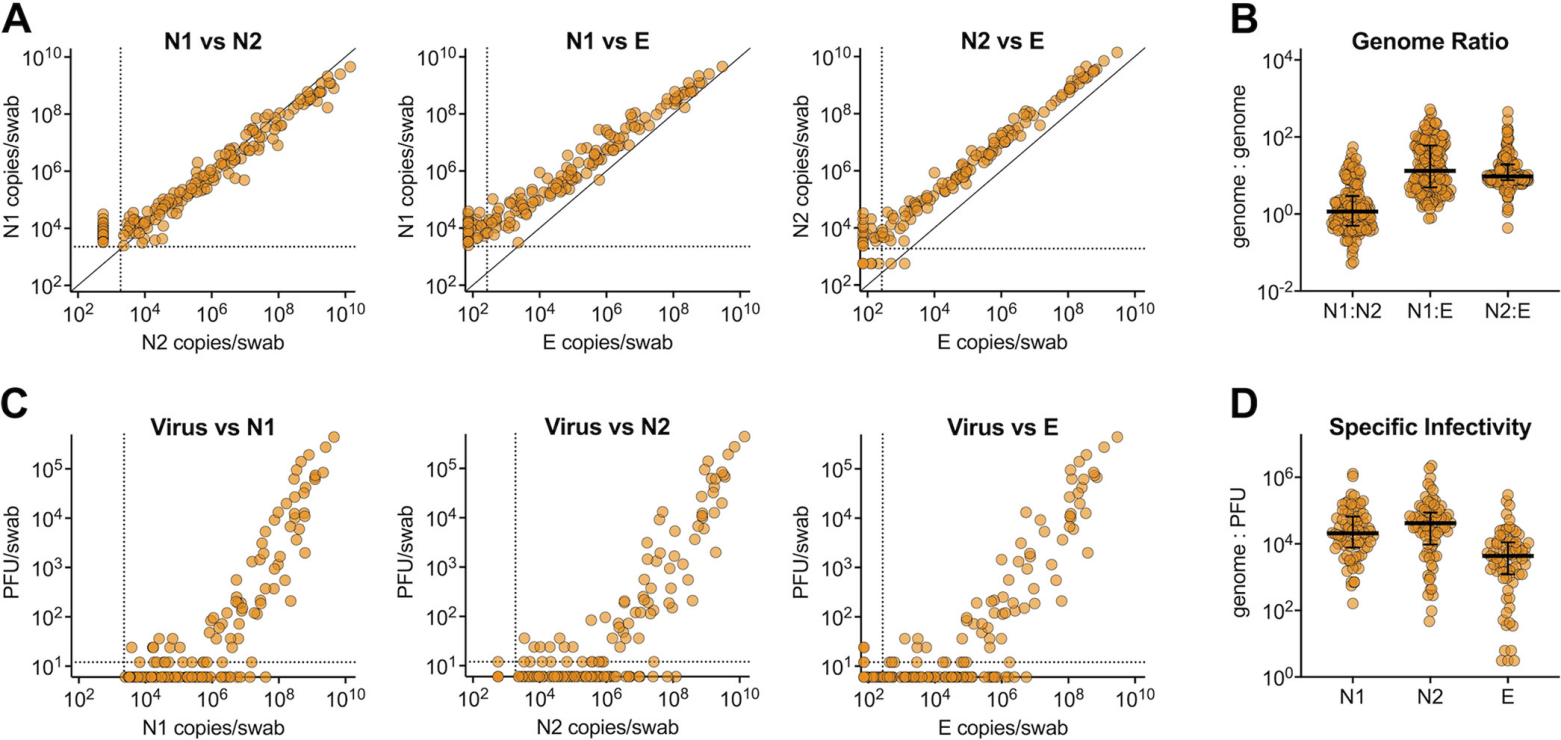
Relationship between SARS-CoV-2 viral RNA and infectious virus. Samples with detectable SARS-CoV-2 N1 vRNA were evaluated for N2 and E vRNA and infectious virus. (A) Relationship between levels of N1, N2, and E vRNA transcripts. (B) Genome/genome ratios between N1/N2, N1/E, and N2/E (median values with interquartile ranges). (C) Relationship between levels of infectious virus and levels of N1, N2, and E vRNA. (D) Specific infectivity (genome/PFU ratio) of infectious virus relative to N1, N2, and E transcripts (median values with interquartile ranges). Dashed lines represent limit of detection.

Swabs with SARS-CoV-2 nucleocapsid region 1 (N1) vRNA were tested for nucleocapsid region 2 (N2) and envelope (E) vRNA-containing viral transcripts (Fig. 2A). We observed high concordance between the levels of N1 and N2 vRNA, with a median genome-to-genome ratio of 1.2 (Fig. 2B). E vRNA levels were lower and less detectable than either N1 or N2 (Fig. 2A), consistent with coronavirus replication, resulting in a higher ratio of N1 and N2 vRNA to vRNA E. (Fig. 2B). Samples with detectable N1 vRNA were also tested for infectious virus. We found a strong positive relationship between vRNA and infectious virus in swab material (Fig. 2C). Infectious virus was rarely detected in individuals with fewer than 10^5^ N1 vRNA copies. However, there were some samples with high levels of vRNA (∼10^7^ copies) with undetectable infectious virus. Virus specific infectivity varied depending on the region of the genome analyzed (Fig. 2D).

### SARS-CoV-2 infection and vRNA levels were not related to age, BMI, sex, or job code.

Age, body mass index (BMI), sex, and smoking habits have been implicated in SARS-CoV-2 infection and disease outcomes (23–29). We detected no significant differences between these variables among vRNA-negative (vRNA^−^) and vRNA-positive (vRNA^+^) individuals (Table 3). Viral RNA levels from N1-positive samples were not dependent on age, BMI, sex, smoking habits, or job code (Fig. S2).

**TABLE 3:** Age, BMI, and smoking status among cohort subsets

Characteristic	Value [mean (range) or % (no. who answered “yes”/no. who answered the question)] for individuals who were^*a*^:		*P* value^*b*^
	vRNA^−^ (*n* = 453)	vRNA^+^ (*n* = 91)	
Age	41 (17–76) (*n* = 453)	41 (16–72) (*n* = 91)	0.7645†
BMI	28.7 (17.8–46.6) (*n* = 190)	28.2 (20.8–43.0) (*n* = 51)	0.3265†
Current smokers	21.2 (40/190)	16.3 (8/49)	0.5516‡
Former smokers^*c*^	20.0 (28/190)	24.5 (12/49)	0.1315‡
Marijuana smokers	5.3 (10/188)	6.1 (3/49)	0.7348‡
Tobacco-based vape product users	6.3 (12/189)	4.2 (2/48)	0.7412‡

a Total numbers for age was available for all individuals of entire cohort, whereas only a smaller subset completed the survey reporting BMI, smoking habbits, and symptoms.

b †, *t* test; ‡, Fisher’s exact test.

c Former smoker refers to those who answered “Yes” to “are you a former smoker?” and “No” to “Do you currently smoke cigarettes?”

### Symptom status differed based on SARS-CoV-2 infection status.

A subset of study participants (*n* = 191 vRNA^−^, *n* = 51 vRNA^+^) responded to a survey to capture their recollection of developing COVID-19-related symptoms before, during, and after the study period (Table 4) (30). All symptoms were significantly more frequent among infected participants. Cough and fever >100.4°F, two symptoms commonly used for COVID screening, were reported in 48% and 24% of infected participants, compared to 14.3% and 7.4% in uninfected individuals. Other symptoms, such as the loss of taste and smell (ageusia and anosmia), were significantly associated with SARS-CoV-2 infection (reported in 2.1% of vRNA-negative and 51.0% of vRNA-positive individuals), consistent with other reports (31).

**TABLE 4:** Symptom status among vRNA-negative and -positive individuals

Symptom	% reporting symptom among individuals who were:		*P* value
	vRNA^−^	vRNA^+^	
Cough	14.3	48.0	<0.001
Dyspnea	8.9	41.2	<0.001
Fever >100.4°F	7.4	24.0	0.0035
Chills/shaking	5.9	40.0	<0.001
Muscle pain	10.6	54.9	<0.001
Headache	22.8	60.8	<0.001
Sore throat	10.7	43.1	<0.001
Ageusia/anosmia	2.1	51.0	<0.001
Diarrhea	5.9	36.0	<0.001
Nasal congestion	16.4	42.0	<0.001
Nausea/vomiting	7.7	25.0	0.002

### Symptom status and severity were related to SARS-CoV-2 infection.

**FIG 3:**
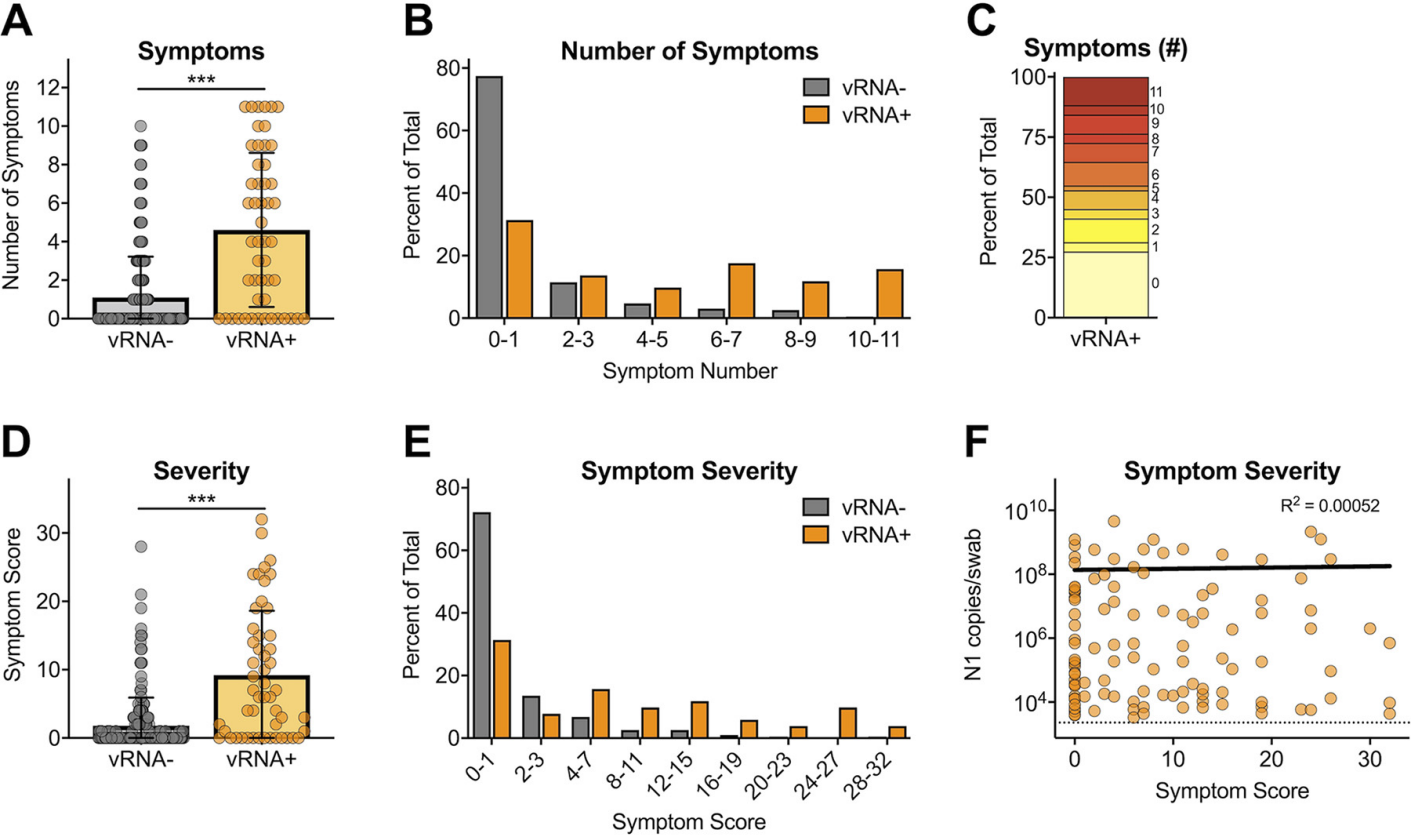
SARS-CoV-2 symptom status, severity, and relationship to viral RNA. (A) Numbers of symptoms reported by vRNA^−^ and vRNA^+^ participants (mean values ± SD). (B) Percentages of vRNA^−^ and vRNA^+^ individuals stratified by number of symptoms. (C) Percentages of vRNA^+^ survey participants reporting total numbers of symptoms. (D) Cumulative symptom score (not reported = 0, mild = 1, medium = 2, severe = 3) for all 11 symptoms stratified by vRNA^−^ and vRNA^+^ participants (mean values ± SD). (E) Percentages of vRNA^−^ and vRNA^+^ individuals stratified by symptom score. (F) Relationship between cumulative symptom score and N1 vRNA levels (semilog nonlinear regression line fit). ***, *P* < 0.0001 by Mann-Whitney unpaired nonparametric test.

More vRNA-positive individuals recalled the development of symptoms than vRNA-negative individuals (*P* < 0.001) (Fig. 3A). Almost 80% of vRNA-negative individuals recalled experiencing 0 to 1 symptoms, whereas vRNA-positive individuals evenly recalled a range of symptoms (Fig. 3B). Twenty-seven percent of vRNA-positive individuals reported never developing symptoms, and 41% reported 2 or fewer symptoms (Fig. 3C). Severity was scored for each symptom (0, no symptom; 1, mild; 2, moderate; 3, severe). A total symptom severity score was calculated by adding the severity scores for each of the 11 symptoms, resulting in a severity score between 0 and 33 for each participant. The severity scores were compared between vRNA-negative and positive individuals. The average symptom severity score was significantly higher in vRNA-positive individuals (*P* < 0.001) (Fig. 3D). Over 70% of vRNA-negative individuals had a symptom severity score of 1 or less, whereas vRNA-positive individuals had an evenly broad range of scores (Fig. 3E). Among vRNA-positive individuals, total symptom scores were not correlated with N1 vRNA levels (Fig. 3F). N1 vRNA levels were stratified by severity for each symptom. N1 vRNA did not predict the severity of any symptom independently (Fig. S3).

### Participants experienced acute, prolonged, and resurgent SARS-CoV-2 infections.

**FIG 4:**
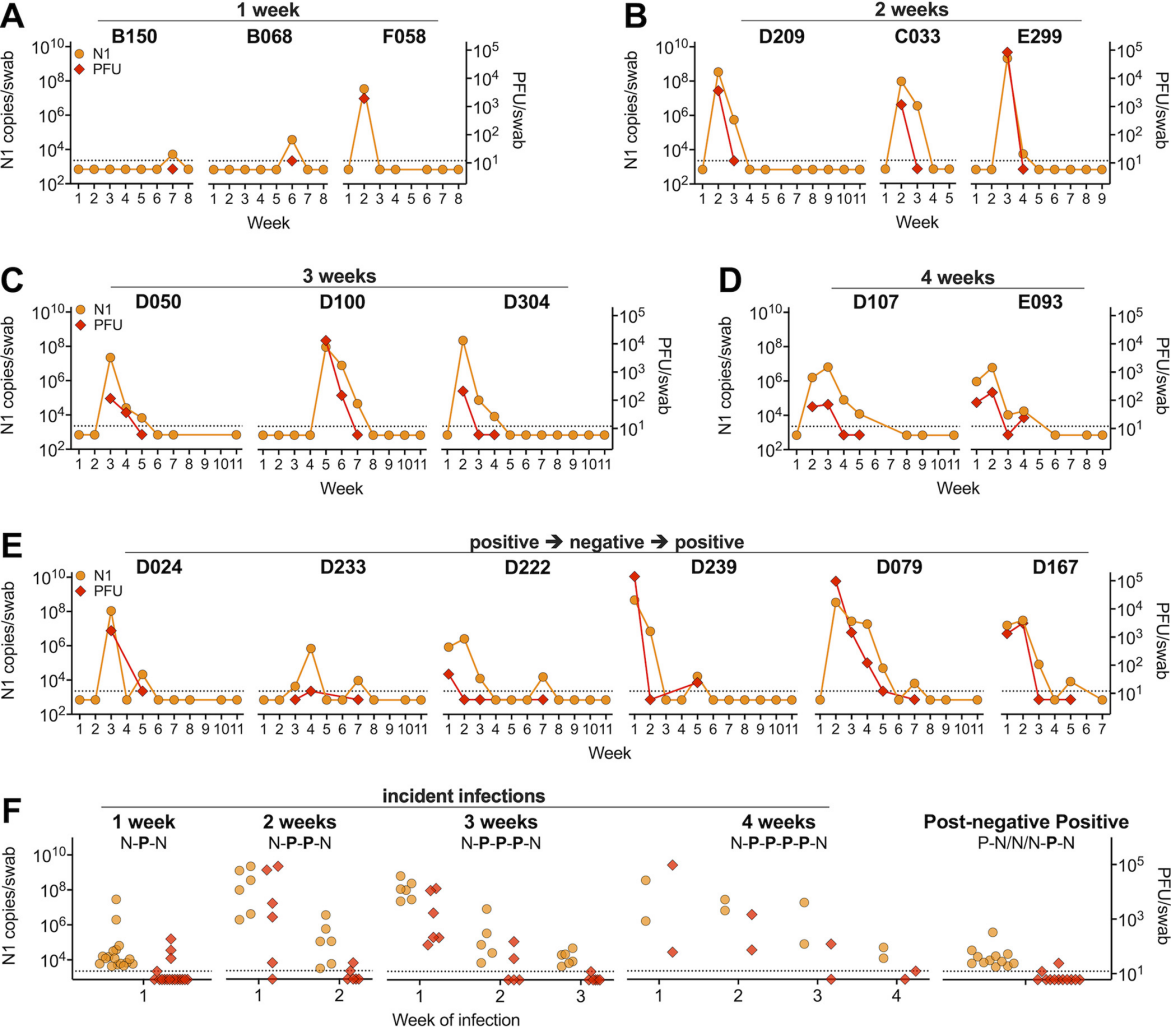
Individual infection courses and virus levels. Viral N1 RNA (left axis) and infectious virus (right axis) in select individuals with detectable N1 for 1 (A), 2 (B), 3 (C), or 4 (D) consecutive weeks. (E) Examples of individuals with detection of N1 vRNA after a period of undetectable N1 following initial infection. (F) N1 vRNA and infectious virus by week of infection are plotted for individuals with incident infections during the course of the study, with negative (N) tests immediately before and after positive (P) tests stratified by the length of infection (1, 2, 3, or 4 consecutive positive weeks) and by those who experienced a postnegative positive test (a positive test after 1 to 3 negative weeks following initial infection). Dashed lines represent limit of detection; samples with result of “not detected” are plotted at half the limit of detection.

Within the cohort and study period, we observed a wide range of infection durations and vRNA loads (Fig. 4A to E). We documented individuals who were positive for a single week with low levels of vRNA and no detectable infectious virus (B150), as well as individuals who tested positive for a single week and had high levels of both vRNA and infectious virus (F058) (Fig. 4A). Individuals who were positive for multiple consecutive weeks often had high levels of vRNA and infectious virus on their first positive test that decreased in subsequent weeks (Fig. 4B to D). There were also individuals with positive SARS-CoV-2 tests followed by 1 to 3 weeks of negative tests before vRNA was again detected (Fig. 4E). Individuals with incident infections during the course of the study, with negative tests before and after positive tests, were stratified based on the number of consecutive vRNA-positive weeks (Fig. 4F). Those who were vRNA positive for a single week tended to have low N1 levels and rarely had infectious virus (Fig. 4F). Virus levels in infections that lasted 2 to 4 weeks were generally highest in the first week and subsequently decreased (Fig. 4F). Individuals with postnegative positive tests (positive after 1 to 3 weeks of negative tests following initial infection) were associated with very low levels of vRNA and rarely infectious virus (Fig. 4F).

### Phylogenetic analysis of SARS-CoV-2 sequences from LTCFs.

**FIG 5:**
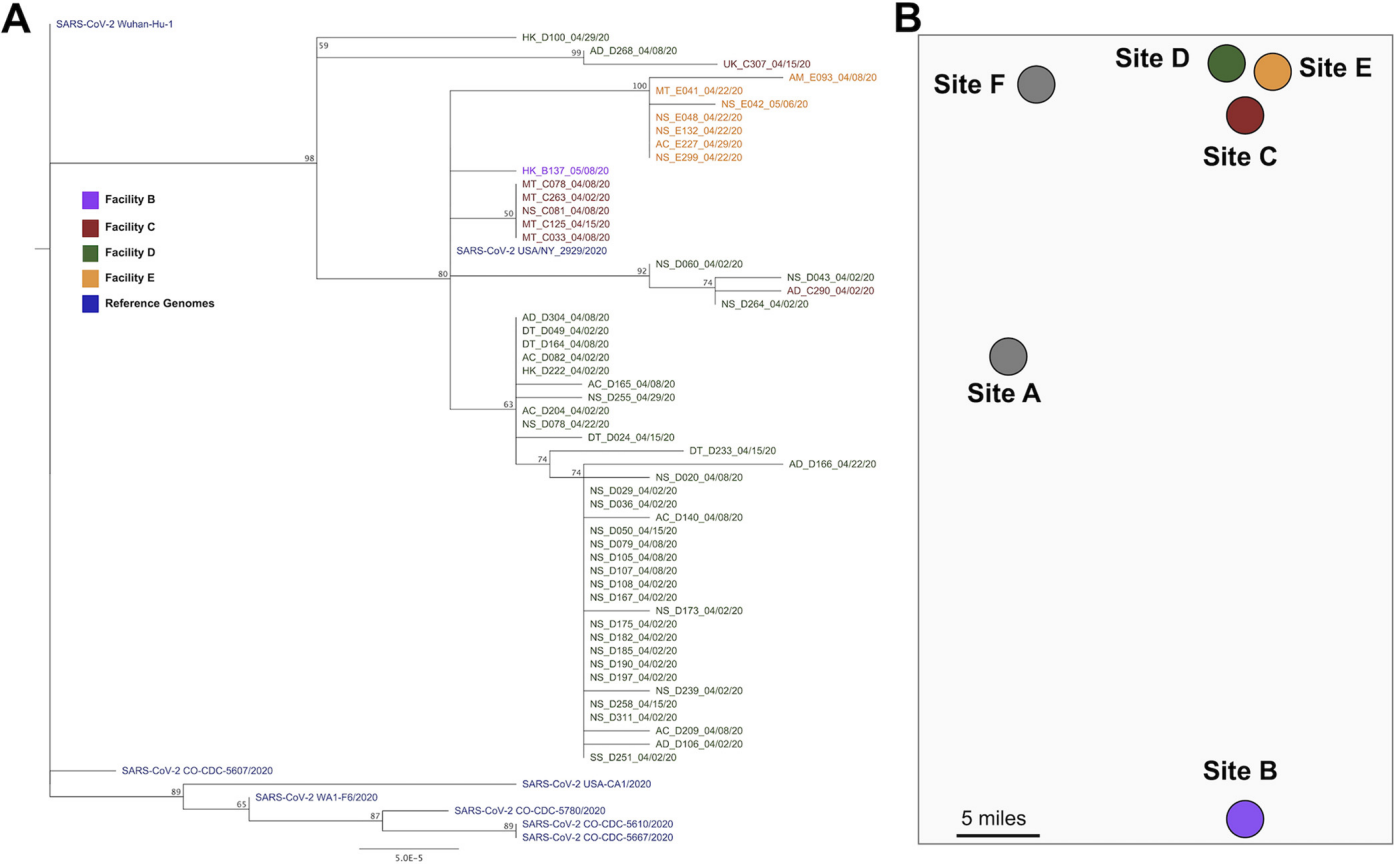
Phylogenetic analysis of SARS-CoV-2 genomes collected from Colorado LTCFs. (A) PhyML tree constructed using Tamura-Nei distance model, including both transitions and transversions, in Geneious Prime. Numbers at nodes indicate bootstrap confidence based on 1,000 replicates. Distance matrix was computed, and the tree was visualized in Geneious Prime. Letters preceding taxon designations represent job codes (AC, activities; AD, administrative; AM, admissions; DT, dietary; MT, maintenance; NS, nursing; SS, social services; UK, unknown), and letters A to E indicate site of origin. Numbers after underscores indicate the date of sample collection. Reference sequences and four Colorado-derived sequences were obtained from NCBI. (B) Map of the LTCFs’ relative geographic locations and distances from one another.

Fifty-four partial genome sequences were obtained from individuals with infections during the study (Fig. 5A). The mean genome coverage was 29,317 nucleotides (nt) (range, 24,076 to 29,835 nt), and the mean coverage depth was 640 reads per position (range, 344 to 2,138 reads). Gaps in sequencing alignment due to ARTIC V2/V3 primer incompatibilities were filled in with the sequence of a reference strain from early in the U.S. outbreak (WA1-F6/2020; accession number MT020881.1). The LTCF sequences were aligned to the sequences of reference strain WA1-F6, four Colorado strains (CO-CDC), and strains from California (USA-CA1), New York (USA/NY), and Wuhan (Wuhan-Hu-1). The tree was reasonably resolved into multiple clusters with moderate bootstrap support (i.e., >50%). The largest cluster is composed exclusively of sequences obtained from individuals at site D (Fig. 5A, lower part of tree). Sequences from sites C (Fig. 5A, red) and E (Fig. 5A, orange) primarily cluster among themselves; however, there are site C sequences within the D clusters as well. The single sequence from site B (B137_05/08/20) is most similar to site C sequences. Sites C, D, and E are all geographically close to one another, whereas site B is ∼50 miles away (Fig. 5B).

## DISCUSSION

LTCFs remain high-risk environments for SARS-CoV-2 transmission (10, 17, 21). Because of their disproportionate contribution to the burden of COVID-19 mortality (2, 3), they also represent an attractive target for continued surveillance (9). Our data clearly demonstrate the potential for large numbers of staff at LTCFs to be asymptomatically/presymptomatically infected and for the concentrations of infections to vary widely across facilities. We documented a significant decline in new infections in facilities with the highest initial infection prevalence following early identification and isolation of SARS-CoV-2-positive staff during the early months of the pandemic. The detection of incident infections at facility B after 3 weeks of negative tests underscores the on-going threat of infections in worker populations, especially in facilities with low vaccination acceptance among staff and/or residents. These results clearly demonstrate that infected staff may be common in specific LTCFs (13–15, 17) and that unvaccinated staff will likely continue to be a reservoir for new outbreaks.

Because coronavirus genome replication creates an abundance of subgenomic N-containing transcripts (32), it is not surprising that higher levels of N transcripts are detected compared to the levels of E vRNA. We found that viral RNA was strongly correlated with infectious virus (samples with high levels of vRNA tended to have high levels of infectious virus, whereas lower vRNA levels often had undetectable levels of infectious virus). Importantly, this demonstrates that individuals with high levels of vRNA are likely infectious to others (33–35). We also detected infectious virus in asymptomatic individuals and at later time points than in other reports, suggesting that the presence and duration of infectious virus varies greatly by the individual (36). It is possible that different viral variants identified in our study have different kinetics or ability to infect cells in culture, leading to slight differences in infectious virus results. Our data support the observation that seemingly healthy staff can harbor high levels of infectious virus in the absence of clinical disease and may therefore contribute to transmission of SARS-CoV-2; thus, continued surveillance is required, particularly for unvaccinated staff.

The impacts of age, sex, BMI, race, ethnicity, and other patient characteristics on SARS-CoV-2 infection and disease outcomes are not well defined (23–28). Within our cohort, we detected no relationship between any of these factors and RNA load, symptom number, or symptom severity. Additionally, while symptom status and severity are strongly correlated with positive SARS-CoV-2 results, viral load is not correlated with either status or severity. Notably, others have found that symptomatic hospitalized patients have lower virus levels than nonhospitalized peers (37). Together, these results suggest that other host or viral factors likely impact virus levels and clinical presentation.

The longitudinal design of this study permitted characterization of individuals’ full infection courses, including those who were positive for 1 to 5 consecutive weeks. In most cases, the viral load was highest in the first week and then declined. Consistent with other reports (38–41), we observed individuals with positive tests after apparent clearance of the initial infection. While it is possible that these individuals were reinfected immediately after clearing their initial infection, we find that unlikely (42, 43). Instead, this may be due to host factors that lead to temporary suppression of virus within the nasopharynx or an improper swab collection that failed to capture sufficient material for detection (44). Importantly, the postnegative positive samples contained low levels of vRNA and low or undetectable infectious virus. These data highlight the heterogeneity of human SARS-CoV-2 infection and the need to further understand host and viral factors that govern infection and clearance.

Virus sequencing provides insights into SARS-CoV-2 transmission (22). Our data encompass 54 genomes obtained from four sites. Strikingly, the viruses primarily cluster by facility, suggesting local transmission among staff at each site. It is possible there are also community-acquired infections which are introduced to the facilities, which could explain data showing similar virus sequences at more than one facility. Alternatively, infected staff who work at multiple facilities may have contributed to the spread of highly related virus strains to more than one facility. Data on the degree of viral genetic diversity in the larger community would add significant power to our ability to discriminate between these two nonmutually exclusive scenarios. Temporal sequencing comparisons would also help elucidate transmission dynamics within facilities and viral evolution among workers within facilities (45).

Overall, our study highlights the high asymptomatic/presymptomatic SARS-CoV-2 infection rates within staff at LTCFs during the early months of the pandemic. Early identification and isolation of these infected and infectious individuals has served as an effective mitigation strategy. The early adoption of voluntary weekly surveillance testing among staff was successful in reducing incident infection in the population of LTCF workers.

## MATERIALS AND METHODS

### Study sites.

Staff at LTCFs provided consent to participate in this study. Nasopharyngeal (NP) swab samples were collected weekly for 8 to 11 weeks. Saliva samples were collected for a single week (week 5) at two facilities (A and B) because nasopharyngeal swabs were unavailable. These two sites were selected for saliva testing over other sites because their SARS-CoV-2 prevalence was 0% in the preceding weeks. Participants provided date of birth and job code but were otherwise deidentified. This study was reviewed and approved by the Colorado State University IRB under protocol number 20-10057H. Participants were promptly informed of test results and, when positive, instructed to self-isolate for 10 days. Individuals that tested positive were instructed to return to the facility the following week for testing. Test results were reported to the appropriate public health authorities. Return to work required absence of fever or other symptoms for the final 3 days of isolation.

### Sample collection.

Nasopharyngeal swabs were collected by trained personnel. Each swab was placed in a conical tube containing 3 ml viral transport medium (Hanks balanced salt solution; 2% fetal bovine serum [FBS], 50 mg/ml gentamicin, 250 μg/ml amphotericin B [Fungizone]). Saliva was collected by repeatedly spitting through a straw into a sterile tube as described by other studies (46, 47).

### RNA extraction.

Tubes containing NP swabs were vortexed and centrifuged to pellet debris. RNA was extracted from supernatant with the Omega Mag-Bind viral DNA/RNA 96 kit using 200 μl of input sample on a KingFisher flex magnetic particle processor according to the manufacturer’s instructions.

### qRT-PCR.

One-step reverse transcription and quantitative PCR (qRT-PCR) was performed using the Express one-step SuperScript qRT-PCR kit (Thermo Fisher Scientific) according to the manufacturer’s instructions. N1, N2, and E primers/probes were obtained from IDT and are described elsewhere (48–50). RNA standards for nucleocapsid (N) and envelope (E) vRNA were provided by Nathan Grubaugh of Yale University and used to determine copy numbers (49). Samples were screened with N1 primers/probes, and those with a cycle threshold (*C_T_*) value of less than 38 were tested for N2 and E vRNA.

### Plaque assay.

Plaque assays were performed on African green monkey kidney (Vero) cells (ATCC CCL-81), which are highly permissive to SARS-CoV-2 and commonly used for plaque assays, according to standard methods (51). Briefly, 250-μl amounts of serially diluted samples were inoculated onto cell monolayers for 1 h. After this initial incubation, cells were overlaid with tragacanth medium, incubated for 2 days, and fixed and stained with 30% ethanol and 0.1% crystal violet. Plaques were counted manually.

### Incidence estimation.

The rate at which staff acquired infections was estimated as the number of new infections per 100 workers per week at each facility from week 2 through the end of the study. Staff were classified as having an incident infection if they tested positive for the first time following a negative test 1 or 2 weeks prior and if they had not previously tested positive for SARS-CoV-2 in our study. The population at risk included all staff who had not yet been infected, to our knowledge, and who tested negative in week 1 of the study.

### Symptom reporting.

Symptom data were collected and managed with REDCap electronic data capture tools hosted at the Colorado Clinical and Translational Sciences Institute (CCTSI) at the University of Colorado Anschutz Medical Campus (52, 53). Survey administrators accessed the survey on a portable tablet computer, entered a participant-specific case number, and provided a verbal introduction. Participants were asked to enter responses to questions concerning symptoms, symptom severity, comorbidities, household size, general characteristics (height, weight, etc.), smoking habits, inhaled medication use, and potential exposure to SARS-CoV-2. Symptom severity and exposure questions were phrased to encompass a range of time from mid-March to late June, which included time before and after our surveillance testing, and therefore, participants might have reported symptoms from infections occurring outside our testing window.

### Next-generation sequencing and analysis.

cDNA was generated using SuperScript IV reverse transcriptase enzyme (Invitrogen) with random hexamers. PCR amplification was performed using ARTIC network V2 or V3 tiled amplicon primers in two separate reactions with Q5 high-fidelity polymerase (NEB) as previously described (45). First-round PCR products were purified using Ampure XP beads (Beckman Coulter). Libraries were prepared using the Nextera XT library preparation kit (Illumina) according to the manufacturer’s protocol. Unique Nextera XT i7 and i5 indexes for each sample were incorporated for dually indexed libraries. Indexed libraries were again purified using Ampure XP beads. The final libraries were pooled and analyzed for size distribution using the Agilent high-sensitivity D1000 ScreenTape on the Agilent TapeStation 2200. Final quantification was performed using the NEBNext library quant kit for Illumina (NEB) according to the manufacturer’s protocol. Libraries were sequenced on the Illumina MiSeq v2 using 2 × 250 paired-end reads.

Sequencing data were processed to generate consensus sequences for each viral sample. MiSeq reads were demultiplexed and quality checked by FastQC, paired-end reads were processed to remove Illumina primers and quality trimmed with Cutadapt, and duplicate reads were removed. The remaining reads were aligned to the SARS-CoV-2 WA1-F6/2020 reference sequence (GenBank accession number MT020881.1) by using Bowtie2. Alignments were further processed and were quality checked using Geneious software, consensus sequences were determined, and any gaps in sequences were filled in with the reference sequence or a cohort-specific consensus sequence. Consensus sequences were aligned in Geneious, and a maximum-likelihood tree generated using PhyML in Geneious with the Wuhan-Hu-1 reference sequence (GenBank accession number MN908947.3) as an outgroup and 100 bootstrap replicates. All sequences are available at GISAID (https://www.gisaid.org/), under accession ID numbers EPI_ISL_527451 - EPI_ISL_527488.

### Statistical analysis.

All statistical analyses were performed using GraphPad Prism version 9.2.0. The Mann-Whitney unpaired nonparametric test was used to compare symptom status and severity. The *t* test was used to compare age and BMI among vRNA-positive and -negative individuals, and Fisher’s exact test was used when comparing smoking and symptom status among vRNA-positive and -negative individuals. The specific statistical tests and *P* values are specified in the figure legends.
