# Supplementary material for: Early Adoption of Longitudinal Surveillance for SARS-CoV-2 among Staff in Long-Term Care Facilities: Prevalence, Virologic and Sequence Analysis
Source: Microbiol Spectr. 2021 Nov 10;9(3):e01003-21. doi: 10.1128/Spectrum.01003-21 (PMC8579921; doi:10.1128/Spectrum.01003-21)
Supplement: SUPPLEMENTAL FILE 1 — Supplemental material. Download SPECTRUM01003-21_Supp_1_seq9.pdf, PDF file, 0.7 MB [file spectrum01003-21_supp_1_seq9.pdf]

Supplemental Legends

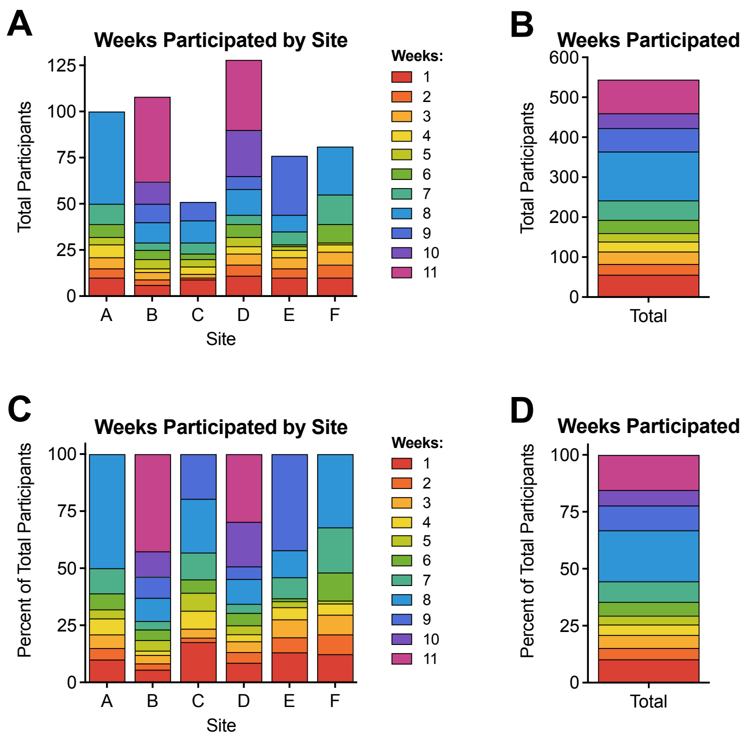

Supplemental Figure 1. Staff participation by week. The number of weeks each staff member participated in testing **A)** by individual site and **B)** in total. The percentage of staff **C)** at each site and **D)** in total that participated by number of weeks.

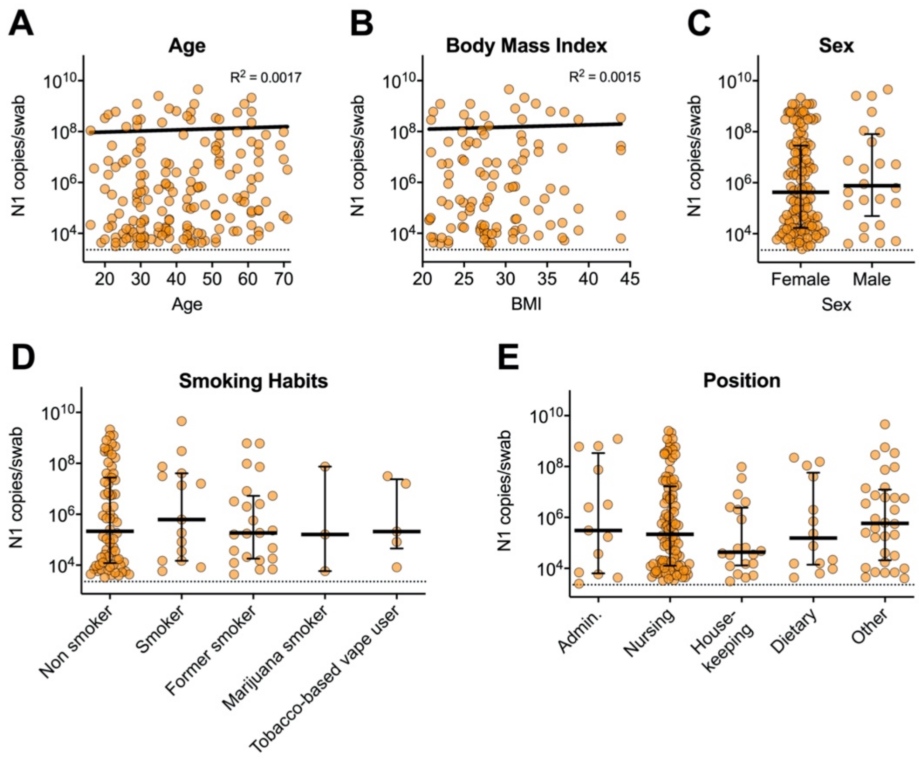

Supplemental Figure 2. Virus levels stratified by participant age, body mass index, sex, and job code. Participants were stratified by **A)** age (n = 91), **B)** BMI (n = 51), **C)** sex (n = 79), **D)** smoking habits, and **E)** job code (n = 90). N1 vRNA from all N1-positive samples were plotted. **A and B)** Semilog nonlinear regression line fit, and **C-D)** bar and errors represent median with interquartile range. Dashed line represents limit of detection.

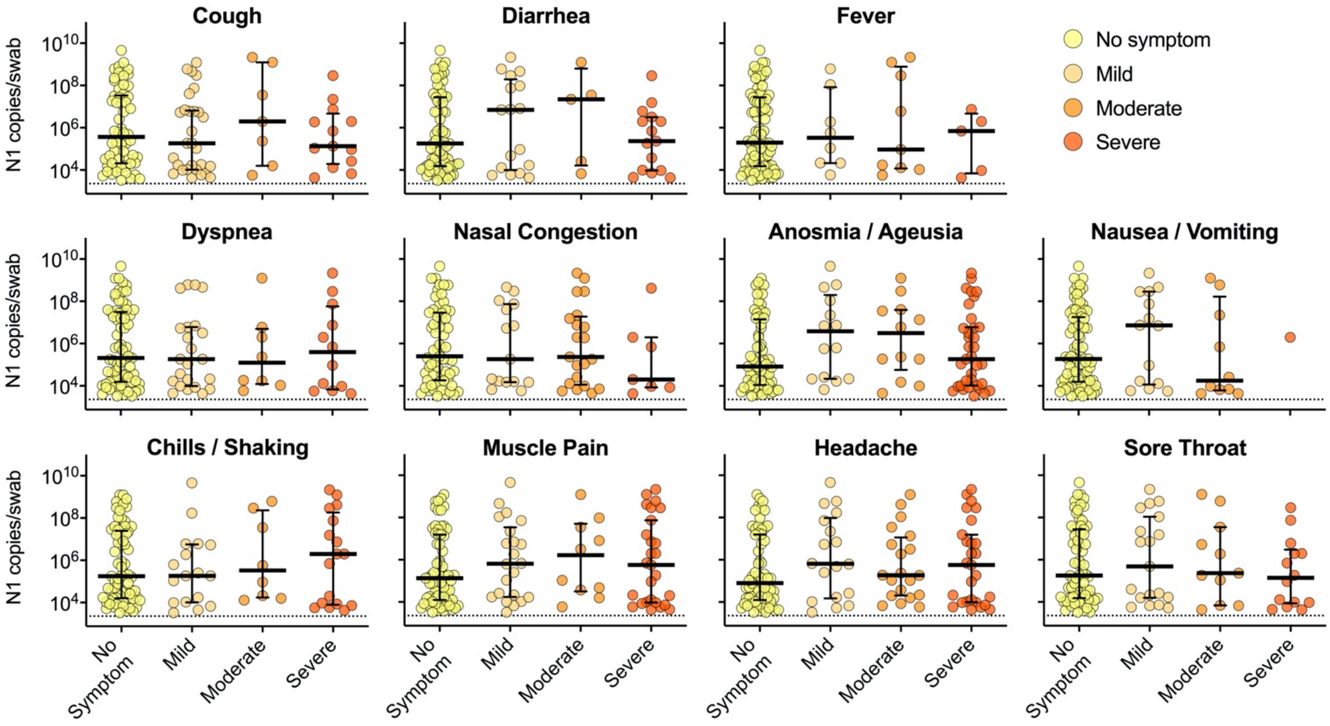

Supplemental Figure 3. N1 vRNA and symptom severity. N1 vRNA levels for each symptom stratified by symptom severity. Bar and errors represent median with interquartile range. Dashed line represents limit of detection.
